# Research priorities for randomised controlled trials in chronic migraine preventive medication: A stakeholder consensus workshop

**DOI:** 10.3310/nihropenres.13548.1

**Published:** 2024-04-04

**Authors:** Sophie Rees, Andrew Cooklin, Callum Duncan, Manjit Matharu, Seyran Naghdi, Martin Underwood, Hema Mistry

**Affiliations:** 1 Bristol Trials Centre, Bristol University, Bristol, BS8 1NU, UK; 2 Patient Representative, UK, UK; 3 Department of Neurology, Aberdeen Royal Infirmary, Aberdeen, Scotland, AB25 2ZN, UK; 4 Queen Square Institute of Neurology and The National Hospital for Neurology and Neurosurgery, University College London, London, England, UK; 5 Warwick Clinical Trials Unit, University of Warwick, Coventry, England, CV4 7AL, UK; 6 Coventry and Warwickshire Partnership NHS Trust, Coventry, England, CV2 2DX, UK

**Keywords:** Chronic migraine, stakeholder workshop, randomised controlled trials

## Abstract

**Background:**

Chronic migraine is a disabling condition that can substantially impact on quality of life. People with chronic migraine have headaches on at least 15 days of every month. Preventative medications aiming to reduce number of days with migraine are available, but high-quality randomised evidence is lacking for many drugs, and it is unclear which medications should be prioritised for research. There is also no existing evidence about patient and clinicians’ priorities for research.

**Methods:**

We undertook a consensus workshop with patient and healthcare professional stakeholders, using nominal group technique, to understand these stakeholders’ priorities for future randomised controlled trials. We reached a consensus on a set of research recommendations for the field.

**Results:**

Eight people with chronic migraine and eleven healthcare professionals took part in an online workshop. Comparisons of calcitonin gene-related peptide monoclonal antibodies (CGRP MAbs) and OnabotulinumtoxinA (BTA) were a top priority for our group. Candesartan and Flunarizine were the top drugs the group wanted to compare against placebo.

**Conclusions:**

These research recommendations should guide researchers in the field, and funders when prioritising commissioned research and assessing funding applications. Particular areas to explore further are Candesartan or Flunarizine versus placebo, and comparing and combining CGRP MAbs with other medications.

## Plain English Summary

Chronic migraine is thought to affect 2–4% of the population. It is a disabling condition that can destroy work and family life. Chronic migraine is defined as headaches on 15 or more days per month, with migraine (e.g. aura, nausea) on at least eight of those days. Drugs can be used to reduce the number of days with headache/migraine. We need more research to know which are most effective and best value for money for the NHS. This will support patients and doctors when making decisions which preventive drugs to use.

We wanted to find out what people with chronic migraine and health professionals think are the most important to study, to help researchers and funders prioritise their work.

We held an online workshop with eight people with chronic migraine and eleven health professionals specialising in headaches. The workshop had four core parts. Firstly, participants were briefed on our research findings regarding our review of the literature on preventative drug treatments. Secondly, the participants were divided into small groups to discuss their top drugs to study against placebo or against other drugs. Each small group had a nominated ‘Facilitator’ to chair the discussion and a notetaker to record it. Thirdly, there was a feedback session from each of the small groups to the whole group. Then finally as a whole group, we asked everyone to vote to rank the top comparisons in order of priority.

The group’s priorities for future research were Candesartan and Flunarizine versus placebo and comparing and testing combinations of drugs. Our findings are useful for researchers and funders in the field of headache.

## Introduction

Migraine is the world’s second commonest disabling disorder ^1^ and the top cause of years lived with disability in people aged 15–49 ^2^. Chronic migraine is defined as headaches on 15 or more days per month for more than three months, with features of migraine (e.g. aura, nausea) on at least eight of those days. Chronic migraine is thought to affect 2-4% of the population ^3, 4^. Those with chronic migraine have the potential to benefit from effective prophylactic drugs to prevent headache days and migraine attacks, and improve quality of life.

Preventive medications can effectively treat chronic migraine, but it can be a challenge for clinicians and patients choosing a medication due to the various side effects and limited evidence. Our 2023 systematic review found that further definitive evidence is needed on many commonly prescribed medications (e.g. amitriptyline, Candesartan, propranolol) ^5– 7^. However, no work exists to guide researchers and funders designing and commissioning research in terms of the priorities of patients and healthcare professionals.

Following completion of systematic reviews of clinical- and cost-effectiveness, and adverse events, we used consensus methodology to translate these findings into useful research recommendations for research priorities, grounded in the perspectives of both people with chronic migraine and headache specialists. Consensus methodology is used in health research to generate research priorities and recommendations ^8– 10^.

In this paper we report on the research recommendations agreed to amongst a group of patient and health professional stakeholders. The findings should guide researchers in the field, and funders when prioritising commissioned research and assessing funding applications.

## Methods

### Design

We used Nominal Group Technique (NGT) ^11^, a method used by health researchers to generate ideas and to facilitate making decisions quickly in a large and diverse group. This method facilitates participation and contribution from all group members. We used small group work to facilitate discussion between patients and healthcare professionals, moderated by experienced facilitators. We designed the workshop and materials together with our Patient and Public Involvement (PPI) representative (AC), who suggested adding a breakaway session, wherein people with migraine and clinicians could meet separately to share thoughts and reflect on any challenges in the mixed groups. This supported patients at the workshop to ensure their voices were heard. At the end of the workshop, participants voted anonymously using an online polling website (Vevox) ^12^. Facilitators were not members of the study team, minimising the possibility of influencing the conversation in any particular direction.

### Sample and recruitment

We aimed to recruit 10 healthcare professionals and 15 people with chronic migraine. We approached people with migraine through the National Migraine Centre’s (NMC) mailing list. Administrators of the NMC sent information about the workshop, a link to the expression of interest form online, and the study team contact details. We used personal networks to approach healthcare professionals directly, aiming for a mix of specialties and backgrounds, such as neurologists, general practitioners with a special interest, and headache nurses. Healthcare professionals were invited to express an interest by contacting the study team.

### The workshop

This project was part of a larger study (Award ID: NIHR132803) which systematically reviewed the literature on preventive drug treatments for chronic migraine ^13^. Having completed those reviews, we sent a summary of the findings to all invitees. The workshop itself took place online using Microsoft Teams and began with a presentation summarising the research and findings of the earlier work packages. We then explained the aims and scope of the workshop and research recommendations. Next, our patient representative spoke about the importance of equal voice within the small groups before the group was split into three breakout ‘rooms’ each containing approximately three people with migraine and four healthcare professionals. Each group also had a member of the study team to take notes (scribe).

The small group work required groups to agree on a top five drug-placebo comparisons and top five drug-drug comparisons. They were tasked to consider:

•     How much evidence we have on the drug

•     Safety (side effects)

•     Efficacy (how effective the drugs were found to be in our study)

•     Feasibility (cost, availability, ease of administration)

To support discussion and decision-making, we provided a crib sheet reporting the key study findings. Participants were reminded that they held valid knowledge and perspectives to bring to the discussion, and they were allowed to suggest comparisons of drugs not included in our study.

Next, the group was split by participant type (all people with migraine in one group and all healthcare professionals in another). With a facilitator, they reflected on the success of/any issues with equal voice in the small group sessions. Scribes provided their group session notes with the rest of the team before the plenary session. As a plenary, we discussed the outcomes from the group work. Voting then took place, followed by a brief discussion of the results and explanation of the team’s next steps and implications for the field. All attendees were provided with a certificate of attendance (health professionals) or thank you letter and payment (people with migraine) by email following the meeting.

### Patient and Public Involvement

Two PPI members of the team were involved in development of this project as co-applicants. One PPI member (AC) continued to provide input throughout the study, and actively contributed to design of the workshop and the materials. For example, AC suggested a separate discussion for all patients in the workshop. AC also provided a great deal of input on the workshop materials to ensure they were accessible and relevant. AC also spoke at the workshop to welcome patients, explain the importance of each participant feeling that they had an equal voice, explained his role within the team and was a notetaker in the small group sessions.

## Results

### Participants

We received 147 expressions of interest in response to the invitation shared by the NMC. Nineteen people were sampled for maximum variation in terms of age, ethnicity, and years living with chronic migraine. Eight people with chronic migraine attended on the day. Fourteen clinicians expressed an interest, and all were invited to the workshop. Eleven attended on the day. Although we invited more people with migraine than health professionals, on the day the balance of attendees was in favour of health professionals. Demographics of our sample is shown in Table 1.

**Table 1.** Consensus workshop attendees demographics.

People with migraine	Characteristic		Number
	Age	18–39	3
		40–59	3
		60+	2
	Ethnicity	White British/Other	4
		Mixed heritage	3
		Asian British/Other	1
	Number of years with CM	0–9	4
		10–20	2
		20+	2
Health professionals	Role	Neurologists	8
		Specialist nurse	1
		GP with special interest	2

### Results of the group work

Each group provided ten top comparisons (five drug vs drug, and five drug vs placebo). We removed duplicate questions to create two lists of top comparisons, which resulted in eleven drug-drug comparisons and eight drug-placebo comparisons for future randomised controlled trials. These are shown in Table 2 and Table 3. Calcitonin gene-related peptide monoclonal antibodies (CGRP MAbs) and OnabotulinumtoxinA (BTA) dominated the top head-to-head drug comparisons identified by the groups, each featuring in six of the eleven comparisons.

**Table 2.** The top drug vs drug comparisons suggested by the small groups (in alphabetical order).

Comparison	Comparator 1	Comparator 2
1	All CGRP MAbs rotation	All CGRP MAbs rotation *
2	BTA + Topiramate	CGRP Mabs
3	CGRP Mabs	BTA
4	CGRP MAbs	CGRP MAbs + gepant
5	CGRP MAbs + BTA	BTA
6	CGRP MAbs + BTA	CGRP Mabs
7	CGRP MAb receptor ^#^	MAb ligand
8	Flunarizine	BTA
9	Melatonin	Amitriptyline
10	Propranolol	BTA
11	Topiramate	Flunarizine

*This meant a study design whereby participants try one CGRP MAb, and if this fails, move on to another, and so on; #Erenumab = a CGRP MAb receptor, all other CGRP MAbs are ligands

**Table 3.** The top drug vs placebo research recommendations suggested by the small groups (in alphabetical order).

Comparison	Comparator 1	Comparator 2
1	Beta-blocker	Placebo
2	Candesartan	Placebo
3	Doxycycline	Placebo
4	Flunarizine	Placebo
5	Melatonin	Placebo
6	Rimegepant	Placebo
7	SNRIs (Duloxetine, Venlafaxine)	Placebo
8	Tricyclic antidepressant	Placebo

### Results of the voting

In the final part of the workshop, participants voted anonymously for their top five choices of the comparisons. The results are shown in Table 4 and Table 5.

We then combined these to make a top 10 and asked participants to rank them in order of priority. The results are shown in Table 6.

**Table 4.** The group's top five drug vs drug comparisons (in order of priority).

Rank	Comparator 1	Comparator 2
1	CGRP MAbs + BTA	CGRP MAbs
2	CGRP MAbs	BTA
3	CGRP MAb receptor	MAb ligand
4	CGRP MAbs + BTA	BTA
5	CGRP MAbs	CGRP MAbs + gepant

**Table 5.** The group's top five drug vs placebo comparisons (in order of priority).

Rank	Comparator 1	Comparator 2
1	Candesartan	Placebo
2	Flunarizine	Placebo
3	Melatonin	Placebo
4	Beta-blocker	Placebo
5	Tricyclic antidepressant	Placebo

**Table 6.** The group's top 10 drug comparisons (in order of priority).

Rank	Comparator 1	Comparator 2
1	CGRP MAbs + BTA	CGRP Mabs
2	Candesartan	Placebo
3	Flunarizine	Placebo
4	CGRP MAbs	BTA
5	CGRP MAbs + BTA	BTA
6	CGRP MAb receptor	MAb ligand
7	Tricyclic antidepressant	Placebo
8	CGRP MAbs	CGRP MAbs + gepant
9	Melatonin	Placebo
10	Beta-blocker	Placebo

## Discussion

This project used consensus methodology to generate research recommendations that are aimed at informing researchers in the field of chronic migraine when designing research projects and funding applications, and to support funders and reviewers assessing funding applications.

Candesartan and Flunarizine were the top drugs the group wanted compared against placebo. The group felt that as there was no evidence for these drugs in our clinical- and cost-effectiveness study, yet these drugs are commonly used to treat chronic migraine, they should be a high priority for research. Candesartan is a cheap and commonly used drug for hypertension, and GPs are familiar with it. In contrast, Flunarizine is not licensed and is more difficult to prescribe. It is currently usually only prescribed through specialist headache services. Researchers should consider this when designing future randomised controlled trial, and it could be argued that Candesartan may be an easier target for changing practice if research found good evidence of its clinical and cost effectiveness in this population.

The list of comparisons initially suggested by the groups included unanticipated drugs such as melatonin and doxycycline. The latter was put forward by one of the small groups, as a group member drew on evidence from a small open label study of four patients ^14^. It was rejected by the wider group and was not included in the final list of priorities. Melatonin was mentioned in more than one of the small groups and was ranked within the top five drug-drug comparisons. This is not a commonly used drug for the management of migraine. One study found it performed better than placebo but not to amitriptyline, but in an episodic migraine population ^15^.

Drugs without good quality evidence (e.g. Candesartan, Flunarizine, tricyclics) were prioritised for drug-placebo comparisons, as there is already good quality evidence for Topiramate, BTA, and MAbs versus placebo. The stakeholder group felt that evidence was needed to understand the differing clinical effectiveness between the different MAbs, MAbs with different targets, and of combining MAbs with BTA versus each alone. We anticipated that the group would prioritise comparing older commonly used drugs with each other, when in fact these drugs were only prioritised for comparison against placebo. Topiramate, BTA and CGRP MAbs do not feature in the top five drug-placebo comparisons, reflecting the existing evidence of the superiority of these medications against placebo.

The group raised the possibility of additive effects of combining medications, which was unanticipated by the study team. Since each of the drugs work through different pathways it is plausible that more substantial effects could be achieved through combinations. Our literature reviews found modest effect sizes, the smallest being 1.49 fewer monthly migraine days for topiramate, and the largest being 2.77 fewer migraine days per month for Fremanezumab ^5^. Effect sizes describe the whole population, including those who did not respond to a medication at all, and so the effect on an individual can be much higher. However, the group felt it is worth exploring if additive effects are possible without negative interaction. For example, if the effects of BTA and a CGRP MAb add up to a mean effect size of 4–5 days reduction in headache or migraine days, that could be transformative for many people with chronic migraine.

## Conclusions

Researchers in the field of preventive migraine medication should consider the research recommendations generated through our stakeholder workshop when designing studies. Particular areas to explore are Candesartan or Flunarizine versus placebo, and comparing and combining CGRP MAbs with other medications.

## Ethics and consent

The study was approved by the University of Warwick Biomedical and Social Sciences Research Ethics Committee (BSREC) on 13 ^th^ February 2023 (Ref: BSREC 49/22-23).

Written informed consent for participation in the study and publication of the participants’ responses was obtained from all participants.

## Data Availability

All data generated or analysed during this study are included in this published article. Further breakdown of this data is unavailable to protect participant confidentiality.
