# Supplementary material for: Research priorities for randomised controlled trials in chronic migraine preventive medication: A stakeholder consensus workshop
Source: NIHR Open Res. 2025 Apr 15;4:16. Originally published 2024 Apr 4. [Version 2] doi: 10.3310/nihropenres.13548.2 (PMC12498513; doi:10.3310/nihropenres.13548.2)
Supplement: Peer review history

# Reviewer report: Reviewer response for version 2

**DOI:** 10.3310/nihropenres.14898.r35413

**Published:** 2025-05-03

**Authors:** Parisa Gazerani

**Affiliations:** 1 Oslo Metropolitan University, Oslo, Norway

This article reports on a stakeholder consensus workshop to identify research priorities for randomized controlled trials (RCTs) in chronic migraine preventive medication. Using the Nominal Group Technique (NGT), the authors engaged patients and healthcare professionals to produce a ranked list of drug comparisons. The study addresses a clear gap in guiding future research priorities based on stakeholder input. The article is clearly written and the study is timely and relevant.

 The research question is important and well-justified.

 The methodology (NGT) is appropriate for generating consensus ideas.

 Patient and Public Involvement (PPI) was meaningfully integrated throughout.

 Presentation of the results is clear, with useful tables summarizing priorities.

 Critical points for improvement:

 Sample size and generalizability:

 The workshop included only 19 participants (8 patients, 11 healthcare professionals).

 The imbalance between patients and professionals may bias the priorities toward clinician perspectives.

 Please expand the limitations section to more explicitly discuss how this small and unbalanced sample may affect the generalizability of the findings.

 Selection bias:

 Participants were mainly recruited via a specific center and networks, potentially limiting the diversity of views.

 It would strengthen the manuscript if the authors acknowledge this more explicitly and discuss its implications.

 Voting process transparency: While rankings of priorities are presented, the actual number of votes or percentages for each option are not shown. Including this information (or explaining why it is not available) would improve transparency and allow readers to assess the strength of consensus.

 Validation of priorities: Only a single round of consensus was conducted without subsequent validation (e.g., second round voting or wider survey). While this is acceptable for an exploratory exercise, the authors should acknowledge that a larger or more iterative process could yield different results.

 Implementation considerations: The conclusions would be strengthened by briefly discussing the feasibility of future trials based on the priorities identified (e.g., regulatory barriers for Flunarizine in the UK).

 International applicability: As the sample is UK-based, it would be helpful if the authors comment on whether they believe the identified priorities are transferable to other healthcare settings.

 Conclusion:

 This is a valuable contribution to research priority setting in chronic migraine. However, addressing the points above would make the article more scientifically sound and transparent, especially concerning sample representation, consensus robustness, and practical implications for implementation.

Is the work clearly and accurately presented and does it cite the current literature?

Yes

If applicable, is the statistical analysis and its interpretation appropriate?

Not applicable

Are all the source data underlying the results available to ensure full reproducibility?

Yes

Is the study design appropriate and is the work technically sound?

Yes

Are the conclusions drawn adequately supported by the results?

Yes

Are sufficient details of methods and analysis provided to allow replication by others?

Yes

Reviewer Expertise:

Clinical research methodology; randomized controlled trials (RCTs); stakeholder engagement in research; health services research; headache disorders (migraine); patient and public involvement (PPI) in research.

I confirm that I have read this submission and believe that I have an appropriate level of expertise to confirm that it is of an acceptable scientific standard, however I have significant reservations, as outlined above.

# Reviewer report: Reviewer response for version 2

**DOI:** 10.3310/nihropenres.14898.r35238

**Published:** 2025-05-03

**Authors:** Hsiangkuo Yuan

**Affiliations:** 1 Thomas Jefferson University, Philadelphia, Pennsylvania, USA

There's no specific response based on my previous comments. This paper describes the research needs of a small group of UK prescribers and patients, addressing an important aspect of comparative drug efficacy. However, it remains unclear what evidence types (RCT, real-world data, or insurance claims) were provided to participants when addressing safety, efficacy, and feasibility questions; additionally, the rationale for excluding gepants from analysis was not discussed. As the study has already been completed, I recognize that certain methodological limitations may not be rectifiable at this stage. Yet, the manuscript would benefit from acknowledging these points in the discussion section to provide appropriate context for the findings while maintaining its overall contribution to the field.

Is the work clearly and accurately presented and does it cite the current literature?

Yes

If applicable, is the statistical analysis and its interpretation appropriate?

Yes

Are all the source data underlying the results available to ensure full reproducibility?

Yes

Is the study design appropriate and is the work technically sound?

Yes

Are the conclusions drawn adequately supported by the results?

Partly

Are sufficient details of methods and analysis provided to allow replication by others?

Yes

Reviewer Expertise:

migraine, outcome, neuroimaging

I confirm that I have read this submission and believe that I have an appropriate level of expertise to confirm that it is of an acceptable scientific standard, however I have significant reservations, as outlined above.

# Reviewer report: Reviewer response for version 2

**DOI:** 10.3310/nihropenres.14898.r35239

**Published:** 2025-05-02

**Authors:** Debashish Chowdhury

**Affiliations:** 1 G B Pant institute of Post Graduate Medical Education and Research, Maulana Azad Medical College, New Delhi, India

I have gone through the author's response and the new version of the manuscript.

 Overall, I am satisfied with the responses and approve then recent version.

Is the work clearly and accurately presented and does it cite the current literature?

Yes

If applicable, is the statistical analysis and its interpretation appropriate?

Not applicable

Are all the source data underlying the results available to ensure full reproducibility?

No source data required

Is the study design appropriate and is the work technically sound?

Yes

Are the conclusions drawn adequately supported by the results?

Yes

Are sufficient details of methods and analysis provided to allow replication by others?

Partly

Reviewer Expertise:

Headache medicine, migraine , trigeminal autonomic cephalalgias

I confirm that I have read this submission and believe that I have an appropriate level of expertise to confirm that it is of an acceptable scientific standard.

# Reviewer report: Reviewer response for version 1

**DOI:** 10.3310/nihropenres.14706.r31875

**Published:** 2024-09-20

**Authors:** Hsiangkuo Yuan

**Affiliations:** 1 Thomas Jefferson University, Philadelphia, Pennsylvania, USA

This paper used the nominal group technique to identify and recommend the top research priority for treating chronic migraine. Eight people with chronic migraine and eleven health professionals participated in this workshop online and concluded the top 10 drug comparisons (in order of priority). The manuscript was well-written, with proper tables. However, here are some comments as follows:

 Introduction:

- For reference #1, please cite the latest YLD paper on migraine.
- For reference #7, please update the citation detail: BMJ Neurology Open 2024, 6(1):e000616.
- The feasibility of the Nominal Group Technique should be described with associated literature.

Methods

- Although a crib sheet was provided, it is uncertain what trial information was involved in the decision-making. Were data from both clinical trials and real-world studies included? Perhaps it would be helpful to provide the crib sheet in the appendix.
- It is uncertain if there’s a predetermined set of comparison questions (vs. open-ended) to choose from.

Results

- There were 2 sets of tables, one from the small group and one from the workshop. Besides melatonin and doxycycline, it is unclear the key differences or similarities between these 2 sets, from the context, results, and implications perspective.
- Table 6 is a combination of table 4 and table 5. Is there a specific reason to show all of them? Also, the ranking from table 4 did not match that from table 6.

Discussion

- For mAb+BTA vs. mAb or BTA, there is real-world data on this. However, it is uncertain whether such real-world data were factored into the consideration.
- I am curious that candesartan ranked 2 ^nd^, even though candesartan is not the most frequently used preventive medication. Could the author provide some explanation?
- Please provide the limitations of this study.

Is the work clearly and accurately presented and does it cite the current literature?

Yes

If applicable, is the statistical analysis and its interpretation appropriate?

Yes

Are all the source data underlying the results available to ensure full reproducibility?

Yes

Is the study design appropriate and is the work technically sound?

Yes

Are the conclusions drawn adequately supported by the results?

Partly

Are sufficient details of methods and analysis provided to allow replication by others?

Yes

Reviewer Expertise:

migraine, outcome, neuroimaging

I confirm that I have read this submission and believe that I have an appropriate level of expertise to confirm that it is of an acceptable scientific standard, however I have significant reservations, as outlined above.

# Response

**Published:** 2025-04-15

**Authors:** Sophie Rees

**Affiliations:** Bristol Trials Centre, Bristol University, Bristol, UK

Introduction:

- For reference #1, please cite the latest YLD paper on migraine.

We have done this

- For reference #7, please update the citation detail: BMJ Neurology Open 2024, 6(1):e000616.

We have done this.

- The feasibility of the Nominal Group Technique should be described with associated literature.

We are unclear what the reviewer like us to add here.  We do not think that there is a need to discuss if it is feasible to use nominal group techniques in health services research.  This is a standard and widely used approach which the current authors have used on multiple occasions.  So far, in 2024, a pubmed search for “nominal group technique” or “nominal group techniques” yields 180 hits. We do not think there is a need to describe in detail the underpinning theory and approach as this is well described in previous literature.

 Methods

- Although a crib sheet was provided, it is uncertain what trial information was involved in the decision-making. Were data from both clinical trials and real-world studies included? Perhaps it would be helpful to provide the crib sheet in the appendix.

The focus in this study was to present data from the systematic review.  We did not include any real world data. We have clarified this in the text. The journal does not allow appendices/supporting material, and so we are unable to provide these.

- It is uncertain if there’s a predetermined set of comparison questions (vs. open-ended) to choose from.

We did not pre-determine comparisons to choose from.

 Results

- There were 2 sets of tables, one from the small group and one from the workshop. Besides melatonin and doxycycline, it is unclear the key differences or similarities between these 2 sets, from the context, results, and implications perspective.

Tables 2 and 3 were generated through the small group discussions. We then discussed these as a large group. Following this, attendees anonymously voted, and these votes produced Tables 4-6. The latter tables represent the comparisons *ranked*, whereas Tables 2-3 are all comparisons of interest listed. They are all shown to demonstrate how we got to our final ‘top 10’ priorities.

- Table 6 is a combination of table 4 and table 5. Is there a specific reason to show all of them? Also, the ranking from table 4 did not match that from table 6.

Table 6 is not a combination of tables 4 and 5. We asked people to rank the 10 comparisons shown in Table 4 and 5. So this is the top 10 in order of priority. We have inserted a figure to more easily display the workshop format which will hopefully clarify this.

 Discussion

- For mAb+BTA vs. mAb or BTA, there is real-world data on this. However, it is uncertain whether such real-world data were factored into the consideration.

We have clarified that we only presented evidence from the randomised controlled trials identified in our review.  All the participants brought their own knowledge and experience to the session

  

- I am curious that candesartan ranked 2 ^nd^, even though candesartan is not the most frequently used preventive medication. Could the author provide some explanation?

Candesartan was of high interest to our group.  In terms of a research recommendation it may not be surprising that the group prioritised a well understood drug with a good safety profile, that is not widely used,  which some believe to effective for which there is no RCT evidence .  It is beyond the scope of this paper to explore the groups’ decision making processes.

  

- Please provide the limitations of this study.

We have added this to the discussion.

# Reviewer report: Reviewer response for version 1

**DOI:** 10.3310/nihropenres.14706.r31709

**Published:** 2024-06-18

**Authors:** Raquel Gil-Gouveia

**Affiliations:** 1 Hospital da Luz Headache Center,, Lisbon, Portugal

Thank you for the opportunity to review this manuscript. However, I do have several concerns about it.

 Major Concerns

 1. **Concept and Objective**

 The main objective is to provide recommendations for research priorities grounded in the perspectives of relevant stakeholders, identified as both people with chronic migraine and headache specialists. 

 However, the most relevant stakeholders for research are primarily researchers, including those in academic institutions, clinical researchers in hospitals, and pharmaceutical companies, as they have the resources and willingness to conduct clinical trials.

 Additionally, governments, policy makers (or insurance companies, depending on the context), and regulatory agencies are crucial stakeholders.

 Healthcare professionals, including neurologists, headache specialists, general practitioners, pharmacists, nurses and patients and patient advocacy groups (but also employers and families of people living with migraine), also play an important role.

 Most of these key stakeholders were not included in the study, which is a significant oversight.

 2. **Patient Bias and Information**

- Patients may struggle to make unbiased decisions about pharmacological agents due to their limited information, which is likely based only on the summary of findings and the presentation given beforehand.
- Healthcare professionals have a different, more informed perspective due to their knowledge and experience with these drugs.
- Patients, on the other hand, might have a biased perspective influenced by their personal experiences with specific medications.

3. **Research Priorities**

- The manuscript does not address important issues related to drug research for migraines, such as safety concerns, combination therapies, dosing strategies, patient adherence, and personalized medicine strategies, including treatments for refractory migraine patients. I find it hard to believe that these topics did not come up in the group discussions.

4. Methodological Concerns

- Small Sample Size: The aim was to recruit 10 healthcare professionals and 15 people with chronic migraine. This relatively small sample size might limit the generalizability of the findings.
- Recruitment Strategy
- Selection Bias: Relying on the National Migraine Centre’s (NMC) mailing list may introduce selection bias, as it may not represent the broader population of people with chronic migraine.
- Convenience Sampling: Using personal networks to recruit healthcare professionals could lead to convenience sampling, potentially biasing the sample towards those with a particular interest or relationship with the researchers.
- Diversity of Participants
- Lack of Diversity: There’s no mention of efforts to ensure diversity among participants, although the sample seems balanced by age and ethnicity. However, there is no information about gender, severity of migraine, number of treatment failures or exposure to the evaluated drugs, and geographic location for people with migraine. For healthcare professionals, age, gender, and years of experience would also be important to ensure diversity.
- Recruitment Communication
- Communication Details: The description does not specify how potential participants were informed about confidentiality, voluntary participation, and the potential risks and benefits of participating in the study.

5. Workshop Concerns

- Online Format
- Access Issues: Conducting the workshop online via Microsoft Teams may exclude participants who lack access to technology or are not comfortable using digital platforms, potentially biasing the sample.
- Group Composition
- Imbalance: Each breakout room had a mix of three people with migraine and four healthcare professionals. This imbalance might affect the dynamics and ensure equal voice, potentially leading to dominance by healthcare professionals.
- Facilitation
- Training Details: The document mentions that a patient representative spoke about the importance of equal voice but does not detail how facilitators were trained to manage group dynamics effectively or address power imbalances.
- Decision-Making Process
- Consensus Building: The criteria for agreeing on top drug comparisons are clear, but the process for resolving disagreements or ensuring consensus within groups is not specified.
- Data Collection
- Standardization: The role of the scribes in note-taking is mentioned, but it’s unclear how the notes were standardized to ensure consistency and reliability across different groups.
- Splitting by Participant Type
- Reinforcing Hierarchies: Separating people with migraine and healthcare professionals for reflection may reinforce hierarchical distinctions, potentially undermining the principle of equal voice.
- Voting and Discussion
- Voting Methods: The methods for voting are not described. It's unclear whether it was anonymous, whether participants had equal voting power, and how voting influenced the final decisions.
- Ethical Considerations
- Ethical Approval: The methods section does not mention ethical approval or how ethical considerations (e.g., informed consent, data protection, right to withdraw) were addressed.
- Outcome Measures
- Effectiveness Measurement: The methods for measuring the effectiveness of the workshop in achieving its aims are not described.
- Stakeholder Engagement
- Feedback Integration: There’s a lack of detail on how stakeholder feedback was integrated into the planning and execution of the workshop, ensuring that it met the needs of both healthcare professionals and people with migraine.

These aspects introduce several types of bias in the data that are difficult to oversee when reading the discussion.

Is the work clearly and accurately presented and does it cite the current literature?

Partly

If applicable, is the statistical analysis and its interpretation appropriate?

Not applicable

Are all the source data underlying the results available to ensure full reproducibility?

No

Is the study design appropriate and is the work technically sound?

Partly

Are the conclusions drawn adequately supported by the results?

Partly

Are sufficient details of methods and analysis provided to allow replication by others?

Partly

Reviewer Expertise:

Migraine, Headache, Health services organization and quality, fMRI, PROMs

I confirm that I have read this submission and believe that I have an appropriate level of expertise to confirm that it is of an acceptable scientific standard, however I have significant reservations, as outlined above.

# Response

**Published:** 2024-08-13

**Authors:** Sophie Rees

**Affiliations:** Bristol Trials Centre, Bristol University, Bristol, UK

**Thank you for the opportunity to review this manuscript. However, I do have several concerns about it.**

**Major Concerns**

**1. Concept and Objective**

**The main objective is to provide recommendations for research priorities grounded in the perspectives of relevant stakeholders, identified as both people with chronic migraine and headache specialists.**

**However, the most relevant stakeholders for research are primarily researchers, including those in academic institutions, clinical researchers in hospitals, and pharmaceutical companies, as they have the resources and willingness to conduct clinical trials. Additionally, governments, policy makers (or insurance companies, depending on the context), and regulatory agencies are crucial stakeholders.**

 The purpose of this project was to understand the priorities from the perspective of those living with and managing the condition. We believe that research should be led by clinical need first and foremost. It is of course up to researchers to identify which of the priorities they are able to research at a particular time, and to address methodological questions. We did not want the workshop to be derailed by discussion of the practicalities of these comparisons: we wanted the focus to be based on the evidence we produced through our systematic reviews, combined with the experiential and clinical knowledge of people living with, and/or treating people with, chronic migraine.

**Healthcare professionals, including neurologists, headache specialists, general practitioners, pharmacists, nurses and patients and patient advocacy groups (but also employers and families of people living with migraine), also play an important role. Most of these key stakeholders were not included in the study, which is a significant oversight.**

 Eleven of our 19 participants were healthcare professionals, including specialists, neurologists, and general practitioners. We recruited people with chronic migraine through a patient-led organisation: National Migraine Centre.

**2. Patient Bias and Information**

**Patients may struggle to make unbiased decisions about pharmacological agents due to their limited information, which is likely based only on the summary of findings and the presentation given beforehand.**

**Healthcare professionals have a different, more informed perspective due to their knowledge and experience with these drugs.**

**Patients, on the other hand, might have a biased perspective influenced by their personal experiences with specific medications.**

 We disagree that patients cannot make unbiased decisions about research priorities. It is common when setting research priorities to draw on patient perspectives. Both health professionals and people living with migraine have their particular perspectives, informed by their experiences. We gave the same information to all the participants who attended the workshop.

**3. Research Priorities**

**The manuscript does not address important issues related to drug research for migraines, such as safety concerns, combination therapies, dosing strategies, patient adherence, and personalized medicine strategies, including treatments for refractory migraine patients. I find it hard to believe that these topics did not come up in the group discussions.**

 In the workshop, we aimed to keep participants on track by focusing only on which comparisons (head-to-head or placebo) they thought would be most important to prioritise. It would of course be important for any researchers developing a study about any of the medications to work with patient representatives and health professionals to determine the dosing strategies, combinations, and ways to understand and support treatment adherence.

**4. Methodological Concerns**

**Small Sample Size: The aim was to recruit 10 healthcare professionals and 15 people with chronic migraine. This relatively small sample size might limit the generalizability of the findings.**

 In this study we were seeking to achieve consensus from a group of interested and informed individuals. This is usual in consensus exercise of this nature. We were not seeking to establish the views of the population as whole.  This would have required a different study design.

**Recruitment Strategy**

**Selection Bias: Relying on the National Migraine Centre’s (NMC) mailing list may introduce selection bias, as it may not represent the broader population of people with chronic migraine.**

 Our experience in previous work is that identifying people with chronic headache disorders  from the general population to participate in research studies is extremely challenging.  For example, in the CHESS trial we needed to approach over 31,000 people to ensure we could recruit 736 participants.  Any such approach would also need NHS research ethical approval.  Pragmatically, working with the National Migraine centre allowed us to recruit participants in a timely and efficient manner.

**Convenience Sampling: Using personal networks to recruit healthcare professionals could lead to convenience sampling, potentially biasing the sample towards those with a particular interest or relationship with the researchers.**

  Our experience across multiple studies of this nature is that it can be very challenging to recruit health professionals willing to give up their time for the project.  Any form of random sampling is almost certainly doomed to failure.  Not least because of the challenge of actually finding data for the sampling frame.  The use of personal networks means we were able to recruit participants for the study.

**Diversity of Participants**

**Lack of Diversity: There’s no mention of efforts to ensure diversity among participants, although the sample seems balanced by age and ethnicity. However, there is no information about gender, severity of migraine, number of treatment failures or exposure to the evaluated drugs, and geographic location for people with migraine. For healthcare professionals, age, gender, and years of experience would also be important to ensure diversity.**

 We asked people expressing an interest to provide basic demographic data and used purposive sampling to generate a sample that was diverse in terms of age, ethnicity, and years living with chronic migraine. This was described in the ‘Participants’ section of the paper, but we have also added this to the methods section. Given the small sample, any attempt to include diversity in all of the above characteristics would result in tokenism. We chose to focus on a small number of characteristics we could realistically achieve diversity in.

**Recruitment Communication**

**Communication Details: The description does not specify how potential participants were informed about confidentiality, voluntary participation, and the potential risks and benefits of participating in the study.**

 We have added the following to the paper: *‘Individuals who expressed an interest were provided by email with an information sheet which included detail on confidentiality, voluntary participation, right/how to withdraw, and the potential risks and benefits of participation. These principles were reinforced at the start of the workshop*.’

**5. Workshop Concerns**

**Online Format**

**Access Issues: Conducting the workshop online via Microsoft Teams may exclude participants who lack access to technology or are not comfortable using digital platforms, potentially biasing the sample.**

 While we agree this is a potential limitation, if we had held the workshop in person this would have disadvantaged those who would need to travel further or who had physical disabilities, caring responsibilities, or less ability to pay for travel.  Our previous experience is that that people living with chronic migraine can find it very difficult to commit to all day in person meeting because of the unpredictable nature of their disease.

**Group Composition**

**Imbalance: Each breakout room had a mix of three people with migraine and four healthcare professionals. This imbalance might affect the dynamics and ensure equal voice, potentially leading to dominance by healthcare professionals.**

 This was a result of the numbers of people invited that actually attended on the day. The facilitators were skilled in ensuring everyone in the group had a chance to contribute and address any dominant voices. We also included a session where all people with migraine could reflect on any challenges to their equal voice in the small group discussions.

**Facilitation**

**Training Details: The document mentions that a patient representative spoke about the importance of equal voice but does not detail how facilitators were trained to manage group dynamics effectively or address power imbalances.**

 We chose facilitators who were experienced in facilitating discussions between patients and healthcare professionals, and who had previous experience of facilitating similar consensus workshops.

**Decision-Making Process**

**Consensus Building: The criteria for agreeing on top drug comparisons are clear, but the process for resolving disagreements or ensuring consensus within groups is not specified.**

 In the small groups, we facilitated discussions about the potential drug comparisons, and disagreements were resolved through discussion. Although we asked the groups to come up with five head-to-head and five placebo comparisons, scribes also noted where the group felt strongly that there were others they would want mentioned. When we returned to plenary, these additional comparisons were presented by the scribes and facilitators, and as a whole group we were able to discuss them. The facilitators were skilled in consensus discussions, and the group members were willing to consider other views and to come to a consensus.

**Data Collection**

**Standardization: The role of the scribes in note-taking is mentioned, but it’s unclear how the notes were standardized to ensure consistency and reliability across different groups.**

 We used a pro forma for the scribes to take notes. This included a table to clearly document the final decisions, and a space for notes to document the discussion. We have added this to the paper which now reads ‘ *Each group also had a member of the study team to take notes (scribe) using a template we provided containing a table to clearly document final decisions, and a space for notes to capture the discussion.*’

**Splitting by Participant Type**

**Reinforcing Hierarchies: Separating people with migraine and healthcare professionals for reflection may reinforce hierarchical distinctions, potentially undermining the principle of equal voice.**

 This was suggested by our PPI representative and co-author. It was intended to provide people with migraine with an opportunity and space to reflect on their equal voice in the small groups, and any opinions they felt unable to express.

**Voting and Discussion**

**Voting Methods: The methods for voting are not described. It's unclear whether it was anonymous, whether participants had equal voting power, and how voting influenced the final decisions.**

 In the paper, it reads ‘participants voted anonymously using an online polling website (Vevox).’ All participants votes counted the same, and the final priorities were based solely on the anonymous voting. We have added this to the paper.

**Ethical Considerations**

**Ethical Approval: The methods section does not mention ethical approval or how ethical considerations (e.g., informed consent, data protection, right to withdraw) were addressed.**

 While it is true that ethical approval is not in the methods section, it is included in the ‘Ethics and consent’ section at the end.

 We have added following text to the methods. *‘* *All individuals who expressed an interest were provided by email with an information sheet which included detail on confidentiality, voluntary participation, right/how to withdraw, and the potential risks and benefits of participation. These principles were reinforced at the start of the workshop.*’

**Outcome Measures**

**Effectiveness Measurement: The methods for measuring the effectiveness of the workshop in achieving its aims are not described.**

 We are unclear what the reviewer is expecting here. The aim of the workshop was to arrive at consensus from a group of health care professional and people with chronic migraine on research priorities. We achieved this aim and would therefore consider the exercise has been effective.

**Stakeholder Engagement**

**Feedback Integration: There’s a lack of detail on how stakeholder feedback was integrated into the planning and execution of the workshop, ensuring that it met the needs of both healthcare professionals and people with migraine.**

 Within the ‘Patient and Public Involvement’ section under methods, we outline how people with migraine were involved in the development of workshop materials and planning. Healthcare professionals were also part of the study team and gave input from their perspective.

**These aspects introduce several types of bias in the data that are difficult to oversee when reading the discussion.**

 We hope that our responses have addressed the concerns of the reviewer. We have also added the following text to the discussion to make it clearer that these are the views of the group we recruited: *‘Our participants were able to provide multiple patient and clinician perspectives on research priorities for chronic migraine.  However, it is possible another group might have identified different priorities.*’

# Reviewer report: Reviewer response for version 1

**DOI:** 10.3310/nihropenres.14706.r31879

**Published:** 2024-06-10

**Authors:** Debashish Chowdhury

**Affiliations:** 1 G B Pant institute of Post Graduate Medical Education and Research, Maulana Azad Medical College, New Delhi, India

I have reviewed the original research article by Rees et al on “research priorities for randomised control trials in chronic migraine prevention medication is stakeholder consensus workshop”

 This workshop was undertaken with patient and healthcare professional stakeholders using the nominal group technique. The paper is based on the consensus recommendations for research on randomized controlled trials for chronic migraine prevention.

 The manuscript is well-written and highlights the pertinent points. I have the following observations and queries:

  

1. It appears that for approaching the healthcare professionals, personal networks were used. How was it done? randomly or as a convenience sampling?
2. How was the sampling number decided?
3. Out of 147 expressions of interest in the participants group ,19 people were sampled based on a number of considerations. How was this sampling done?
4. Whether this 19 participants were on any preventive medications and how were they responding to them? This is important to know because this might have influenced their voting choices depending on the clinical response they were getting from a particular preventive.
5. I find it a little intriguing that greater occipital nerve blocks have not been included in the list. There is emerging evidence of its efficacy in chronic migraine patients ( few RCTs are available) and also in combination with oral medications such as topiramate. This is a cheap treatment, easily accessible,  and easy to administer. Hence I think this needs to be explored further.
6. The authors may also add a few lines regarding the ethical dilemma of testing the suggested drugs against a placebo as currently there is reasonable evidence to support the use of Botulinum toxin -A and CGRP mabs in chronic migraine patients. How can these patients be deprived of these drugs and given a placebo for such a disabling disease? Hence, instead of placebo-controlled trials,  non-inferiority design trials would probably be best. Although I agree that the robust evidence comes through a placebo-controlled trial, the ethical dilemma remains.

Is the work clearly and accurately presented and does it cite the current literature?

Yes

If applicable, is the statistical analysis and its interpretation appropriate?

Not applicable

Are all the source data underlying the results available to ensure full reproducibility?

No source data required

Is the study design appropriate and is the work technically sound?

Yes

Are the conclusions drawn adequately supported by the results?

Yes

Are sufficient details of methods and analysis provided to allow replication by others?

Partly

Reviewer Expertise:

Headache medicine, migraine , trigeminal autonomic cephalalgias

I confirm that I have read this submission and believe that I have an appropriate level of expertise to confirm that it is of an acceptable scientific standard, however I have significant reservations, as outlined above.

# Response

**Published:** 2024-08-13

**Authors:** Sophie Rees

**Affiliations:** Bristol Trials Centre, Bristol University, Bristol, UK

**I have reviewed the original research article by Rees et al on “research priorities for randomised control trials in chronic migraine prevention medication is stakeholder consensus workshop”**

**This workshop was undertaken with patient and healthcare professional stakeholders using the nominal group technique. The paper is based on the consensus recommendations for research on randomized controlled trials for chronic migraine prevention. The manuscript is well-written and highlights the pertinent points. I have the following observations and queries:**

**It appears that for approaching the healthcare professionals, personal networks were used. How was it done? randomly or as a convenience sampling?**

 We used purposive sampling to ensure a mix of clinical roles and experience. Everyone who responded expressing an interest was invited to the workshop.

**How was the sampling number decided?**

 The target sample size (10 health professionals and 15 people with migraine) was determined using our previous experience of running similar workshops and the practicalities of including a range of voices but also allowing everyone a chance to contribute. We have added this rationale to the paper.

**Out of 147 expressions of interest in the participants group ,19 people were sampled based on a number of considerations. How was this sampling done?**

 Purposive sampling was used based on their responses to the expression of interest. We aimed for maximum variation in terms of age, ethnicity, and years lived with chronic migraine. This is included in the paper under the ‘participants’ section.

**Whether this 19 participants were on any preventive medications and how were they responding to them? This is important to know because this might have influenced their voting choices depending on the clinical response they were getting from a particular preventive.**

 We did not ask participants to state this information about themselves. Our view is that it would have been inappropriate for a consensus exercise of this nature to ask participants for detailed clinical information of this nature.

**I find it a little intriguing that greater occipital nerve blocks have not been included in the list. There is emerging evidence of its efficacy in chronic migraine patients ( few RCTs are available) and also in combination with oral medications such as topiramate. This is a cheap treatment, easily accessible,  and easy to administer. Hence I think this needs to be explored further.**

 This exercise was specifically designed to consider preventive medications rather than interventional procedures.  We included Botox as a medication as it fits into same part of the care pathway as other preventive medications.

**The authors may also add a few lines regarding the ethical dilemma of testing the suggested drugs against a placebo as currently there is reasonable evidence to support the use of Botulinum toxin -A and CGRP mabs in chronic migraine patients. How can these patients be deprived of these drugs and given a placebo for such a disabling disease? Hence, instead of placebo-controlled trials,  non-inferiority design trials would probably be best. Although I agree that the robust evidence comes through a placebo-controlled trial, the ethical dilemma remains.**

 We presented our participants with the known data for effectiveness of medications.  They discussed whether head-to-head, or placebo-controlled trials were appropriate.  We are reporting on their recommendations.  Discussing the ethical issues of placebo-controlled trials and the role, if any, of non-inferiority studies in this area is beyond the scope of this paper.  Our view is that this would need a fresh piece of empirical work to assess acceptability.  Although of course the objective evidence on acceptability would come from testing recruitment to placebo-controlled trials.
